# Supplementary material for: Unusual Patterns of Lateral Scutes in Two Olive Ridley Turtles and Their Genetic Assignment to the Thai Andaman Sea Populations of Lepidochelys olivacea Eschscholtz, 1829
Source: Biology (Basel). 2024 Jul 4;13(7):500. doi: 10.3390/biology13070500 (PMC11273376; doi:10.3390/biology13070500)
Supplement: Supplementary file 1 [file biology-13-00500-s001.zip › Table S1.pdf]

Table S1. The accession numbers of all *Lepidochelys* species used for phylogenetic tree in this study.

Accession number	Species	Reference
OP821911-OP821927	*Lepidochylys olivacea*	Martín-del-Campo, et al. [28]
MW221467-MW211476	*Lepidochylys olivacea*	Warallanda, et al. [29]
OP716909-OP716914	*Lepidochylys olivacea*	Castillo-Morales, et al. [30]
MN342235-MN342242	*Lepidochylys olivacea*	Stelfox, et al. [31]
JN391445-JN391465	*Lepidochylys olivacea*	Jensen and Nancy [32]
KF385935-KF385942	*Lepidochylys kempii*	Frey, et al. [33]
MN159143-MN159152	*Lepidochylys kempii*	Frandsen, et al. [27]
MZ043566-MZ043572	*Lepidochylys kempii*	Lamont, et al. [34]

References

27. Frandsen, H.R.; Figueroa, D.F.; George, J.A. Mitochondrial genomes and genetic structure of the Kemp's ridley sea turtle (Lepidochelys kempii). *Ecology and Evolution* **2020**, *10*, 249-262.

28. Martín-del-Campo, R.; Ortega-Ortiz, C.D.; Abreu-Grobois, A.; Enríquez-Paredes, L.M.; Petatán-Ramírez, D.; García-Gasca, A.; Quijano-Scheggia, S.I. Genetic Evidence for Indo-Western Pacific Olive Ridley Sea Turtles in Mexican Waters. *Diversity* **2023**, *15*, 430.

29. National Center for Biotechnology Information. National Center for Biotechnology Information. **2022**. MW221467-MW211476. Available online: https://www.ncbi.nlm.nih.gov/popset?DbFrom=nuccore&Cmd=Link&LinkName=nuccore_popset&IdsFromResult=2216793848. *accessed on 25 January 2024*.

30. Castillo-Morales, C.A.; Sáenz-Arroyo, A.; Castellanos-Morales, G.; Ruíz-Montoya, L. Mitochondrial DNA and local ecological knowledge reveal two lineages of leatherback turtle on the beaches of Oaxaca, Mexico. *Scientific Reports* **2023**, *13*, 8836.

31. Stelfox, M.; Burian, A.; Shanker, K.; Rees, A.F.; Jean, C.; Willson, M.S.; Manik, N.A.; Sweet, M. Tracing the origin of olive ridley turtles entangled in ghost nets in the Maldives: A phylogeographic assessment of populations at risk. *Biological conservation* **2020**, *245*, 108499.

32. National Center for Biotechnology Information. National Center for Biotechnology Information

2013. JN391445-JN391465. Available online: https://www.ncbi.nlm.nih.gov/popset?DbFrom=nuccore&Cmd=Link&LinkName=nuccore_popset&IdsFromResult=399658696 *accessed on 25 January 2024*.

33. Frey, A.; Dutton, P.H.; Shaver, D.J.; Walker, J.S.; Rubio, C. Kemp’s ridley Lepidochelys kempii nesting abundance in Texas, USA: a novel approach using genetics to improve population census. *Endangered Species Research* **2014**, *23*, 63-71.

34. Lamont, M.M.; Moreno, N.; Camacho-Sánchez, F.Y.; Acosta-Sánchez, H.H.; Glaberman, S.; Reyes-Lopez, M.A.; Chiari, Y. Genetic diversity of immature Kemp's ridley (Lepidochelys kempii) sea turtles from the northern Gulf of Mexico. *Aquatic Conservation: Marine and Freshwater Ecosystems* **2021**, *31*, 3003-3010.
