# Supplementary material for: Unusual Patterns of Lateral Scutes in Two Olive Ridley Turtles and Their Genetic Assignment to the Thai Andaman Sea Populations of Lepidochelys olivacea Eschscholtz, 1829
Source: Biology (Basel). 2024 Jul 4;13(7):500. doi: 10.3390/biology13070500 (PMC11273376; doi:10.3390/biology13070500)
Supplement: Supplementary file 1 [file biology-13-00500-s001.zip › Table S2.pdf]

**Table S2.** Percent identity of the individual 1 and 2, based on the Basic Local Alignment Search Tool (BLAST) available at the National Center for Biotechnology Information (NCBI) GenBank, of D-loop.

| Sample name | BLAST result | Percent identity | Accession number |
|---|---|---|---|
| Individual 1 (Sample GD) | *Lepidochelys olivacea* | 100.00 | MN342242.1 |
| | *Lepidochelys olivacea* | 100.00 | MN342241.1 |
| | *Lepidochelys olivacea* | 99.81 | MN342237.1 |
| | *Lepidochelys olivacea* | 99.81 | MN342236.1 |
| | *Lepidochelys olivacea* | 99.81 | MN342235.1 |
| | *Lepidochelys olivacea* | 99.63 | OP821927.1 |
| | *Lepidochelys olivacea* | 98.51 | MW221485.1 |
| | *Lepidochelys olivacea* | 97.90 | MN535096.1 |
| | *Lepidochelys olivacea* | 97.90 | MH375632.1 |
| | *Lepidochelys olivacea* | 97.90 | MH375631.1 |
| | *Lepidochelys kempii* | 95.41 | MH136061.1 |
| individual 2 (Sample HT) | *Lepidochelys olivacea* | 99.81 | JN391463.1 |
| | *Lepidochelys olivacea* | 99.64 | MN535096.1 |
| | *Lepidochelys olivacea* | 99.64 | MH375632.1 |
| | *Lepidochelys olivacea* | 99.64 | MH375631.1 |
| | *Lepidochelys olivacea* | 99.62 | KM357632.1 |
| | *Lepidochelys olivacea* | 99.62 | KM357630.1 |
| | *Lepidochelys olivacea* | 99.62 | KM357629.1 |
| | *Lepidochelys olivacea* | 99.61 | MW221476.1 |
| | *Lepidochelys olivacea* | 99.61 | MW221475.1 |
| | *Lepidochelys olivacea* | 99.61 | MW221474.1 |
| | *Lepidochelys kempii* | 94.29 | MZ043570.1 |
